# Risk of Autoimmune Diseases in Patients With Interstitial Cystitis/Bladder Pain Syndrome: A Nationwide Population-Based Study in Taiwan

**DOI:** 10.3389/fmed.2021.747098

**Published:** 2021-09-20

**Authors:** Hann-Ziong Yueh, Min-Hsin Yang, Jing-Yang Huang, James Cheng-Chung Wei

**Affiliations:** ^1^Department of Medical Education, Chung Shan Medical University Hospital, Taichung, Taiwan; ^2^Institute of Medicine, College of Medicine, Chung Shan Medical University, Taichung, Taiwan; ^3^Department of Urology, Chung Shan Medical University Hospital, Taichung, Taiwan; ^4^Department of Medical Research, Center for Health Data Science, Chung Shan Medical University Hospital, Taichung, Taiwan; ^5^Department of Allergy, Immunology and Rheumatology, Chung Shan Medical University Hospital, Taichung, Taiwan; ^6^Graduate Institute of Integrated Medicine, China Medical University, Taichung, Taiwan

**Keywords:** autoimmune diseases, interstitial cystitis, bladder pain syndrome, cohort study, nationwide population

## Abstract

**Objective:** The association between autoimmune diseases (ADs) and interstitial cystitis/bladder pain syndrome (IC/BPS) has long been investigated. However, the lack of comprehensive descriptions of patients in the literature has made comparison and evaluation impossible. We aim to investigate the risk of systemic ADs in patients with IC/BPS in Taiwan using a population-based administrative database.

**Methods:** This study evaluated 1,095 patients newly diagnosed with IC/BPS between 2000 and 2013, using data from Taiwan's National Health Insurance Research Database. These patients were randomly matched by demographic characteristics with a comparison cohort of individuals without IC/BPS at a ratio of 1:20. Cox proportional hazards regression analysis was used to analyze the risk of ADs, adjusting for age, sex, urbanization, length of hospital stay, and comorbidities adjustment. Sensitivity analysis by propensity score was used to adjust for confounding factors.

**Results:** The adjusted Hazard Ratio (aHR) of ADs for IC/BPS patients was 1.409 (95% CI 1.152–1.725). The subgroup analysis indicated that female or 45–60 years of age had a greater risk of ADs. Furthermore, the subgroup analysis of primary outcomes indicated that IC/BPS had greater incidence with Hashimoto's thyroiditis (aHR = 2.767, 95% CI 1.039–7.368), ankylosing spondylitis (aHR = 2.429, 95% CI 1.264–4.67), rheumatoid arthritis (aHR = 1.516, 95% CI 1.001–2.296), and Sjogren's syndrome (aHR = 1.962, 95% CI 1.37–2.809).

**Conclusion:** IC/BPS was associated with the development of ADs in our study population, especially Hashimoto's thyroiditis, ankylosing spondylitis, rheumatoid arthritis, and Sjogren's syndrome. Clinicians are recommended to be alert to the increased likelihood of developing ADs, particularly for middle-aged women.

## Introduction

Interstitial cystitis/bladder pain syndrome (IC/BPS) is a chronic inflammatory disease characterized by recurrent pain, discomfort, or tenderness in the urinary bladder and pelvic region and can be accompanied by various urinary symptoms, such as urinary frequency, persistent urge to void, and nocturia. The overall prevalence of IC/BPS is roughly 300 cases per 10^5^ patients, with a five times higher incidence in women than men (1). IC/BPS has multiple possible etiologies that include (1) infection, (2) neuronal function change, (3) autoimmune reactivity, (4) hypersensitivity response with mast cell release, and (5) defects in the urothelial permeability barrier (2). Other confusable diseases with presentations similar to IC/BPS must be ruled out through proper examination before diagnosis (2).

Autoimmune diseases (ADs), although considered to occur infrequently, substantially influence mortality and morbidity of patients. The association between autoimmune reactivity and IC/BPS has long been investigated. Some studies have reported on autoantibodies in patients with IC/BPS (3–5). Kujala et al. proposed a possible link between ADs and IC/BPS in patients with certain immunological factors or genetic predispositions (6). Numerous studies on autoantibodies against nuclear or bladder epithelium antigens in patients with IC/BPS appear in the literature (7, 8). Other studies have also proposed activation of complement components (9). Although the precise identities of relevant autoantibodies have yet to be determined, some speculate that the increased prevalence of ADs among patients with IC/BPS may involve the presence of shared underlying autoimmune disturbances.

In previous population-based studies, patients with IC/BPS had an increased prevalence of several ADs, including rheumatoid arthritis (RA), systemic lupus erythematosus (SLE), Sjögren syndrome (SS), ankylosing spondylitis (AS), and inflammatory bowel syndrome (10–13). The correlation between IC/BPS and other ADs has also been described in several retrospective articles (7, 14, 15). However, the literature is limited due to the use of varying definitions of IC/BPS and approaches to cross-sectional study design. Furthermore, to date, no comprehensive studies have investigated systemic ADs in patients with IC/BPS. These limitations have made comparison and evaluation impossible. To overcome these limitations, we designed a cohort study using a nationwide population database to investigate the risk of systemic ADs in patients with IC/BPS. Furthermore, we performed subgroup analysis and sensitivity testing for further evaluation of the relationship between these two diseases.

## Methods

### Data Source

This population-based retrospective cohort study was constructed using Taiwan's National Health Insurance (NHI) Research Database (NHIRD), which was established in 1995. More than 99% of the population in Taiwan is covered under the NHI program. All medical claims for insured services reimbursement are contained in the NHIRD. Original claims data include inpatient stays, outpatient visits, ambulatory and emergent care, hospitalization records, and medicine prescriptions. The Longitudinal Health Insurance Database, a subset of the NHIRD comprising 1,000,000 systematically and randomly selected beneficiaries from the NHIRD, was used in this study. The diagnostic codes were defined according to the *International Classification of Diseases, Ninth Revision, Clinical Modification* (ICD-9-CM) codes. All identification data were encrypted to protect patient privacy. This study was approved by the Institutional Review Board of Chung Shan Medical University (IRB CS15134).

### Participants

**Figure 1:**
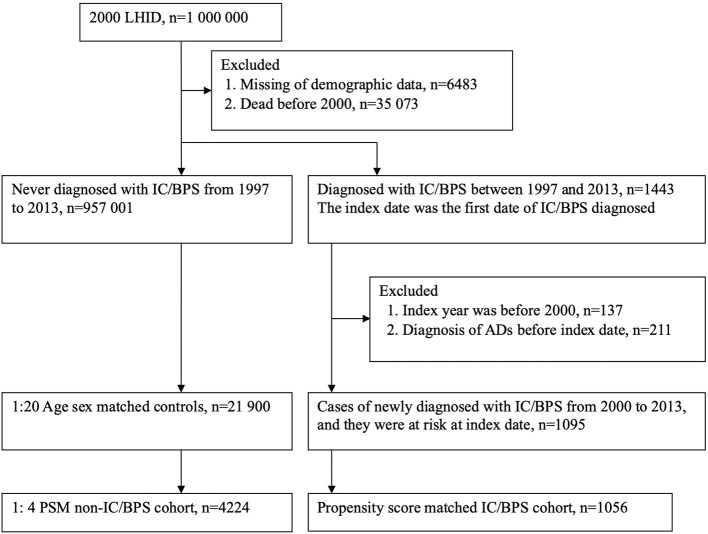
Study flow chart for population selection. LHID, Longitudinal Health Insurance Datasets; IC/BPS, interstitial cystitis/bladder pain syndrome; ADs, autoimmune diseases; PSM, propensity score matching.

For the period 1997 to 2013, we identified patients with new onset of IC/BPS by using ICD-9-CM code 595.1, who had at least two outpatient or one inpatient diagnosis of IC/BPS. The date of first outpatient visit or admission with an ICD-9-CM code for IC/BPS was defined as the index date for the cohort study. To increase the diagnostic validity of IC/BPS, each patient in the study group achieved the prerequisite to use sodium hyaluronate (Cystistat), which works as an intravesical instillation therapy of IC/BPS. According to the NHI regulations, a strict pre-review of medical records was required to prescribe Cystistat for patients with IC/BPS. To date, numerous published studies have been conducted to assure the enrollment of IC/BPS (16–18). Initially, 1443 patients diagnosed with IC/BPS were identified. In order to enroll patients with new-onset IC/BPS to analyze the risk of ADs, we excluded those patients who had been diagnosed with IC/BPS before January 1, 2000 (*n* = 137). Additionally, diagnoses of ADs before the index date were excluded (*n* = 211). Individuals without IC/BPS were individually paired at a 1:20 ratio with IC/BPS patients based on age and sex at the index date. In total, 1,095 patients with IC/BPS and 21,900 persons without IC/BPS were selected for analysis (Figure 1). All individuals enrolled in the study were tracked from the index date until the development of ADs, withdrawal from the NHI program, death, or the end of the study (December 31, 2013).

### Study Outcomes

Occurrence of ADs was defined as the primary outcome when subjects had at least 2 outpatient or 1 inpatient diagnosis of a condition listed below. These codes were validated and published in previous studies (19–21). The subcategories of ADs include Graves' disease (ICD-9-CM code 242.0), Crohn's disease (555), psoriasis and similar disorders (696), SLE (710.0), RA (714), AS (720.0), Guillain–Barré syndrome (357.0), SS (710.2), myasthenia gravis (358.0), vasculitis (446), uveitis (360.12, 363.0×, 363.1×, 363.20, 363.21, 363.22, 364.0×, 364.1×, 364.2×, 364.3), polymyalgia rheumatica (725), dermatomyositis (710.3), Hashimoto's thyroiditis (245.2), Behcet's disease (136.1), polymyositis (710.4), ulcerative colitis (556), autoimmune hemolytic anemia (283.0), multiple sclerosis (340), and systemic sclerosis (710.1). The diagnoses of ADs are highly trustworthy because systemic ADs such as SLE, SS, and RA are rigorously verified by two certified rheumatologists to review patients' medical records before issuing a catastrophic illness certificate, which is requested by the financial entity from the Bureau of NHI bearing the medical copayments of the patient (10, 12, 22).

### Comorbidities

We considered comorbidities associated with ADs that should be included when adjusting for potential confounding factors. The following comorbidities were included as study covariates: hypertension (ICD-9-CM codes 401-405), type 2 diabetes mellitus (DM; 250), hyperlipidemia (272), coronary artery disease (CAD; 410-414), stroke (430-438), chronic kidney disease (CKD; 403.11, 403.91, 404.12, 404.13, 404.92, 404.93, 585, 586, 587, 274.1, 403.10, 403.90, 404.10, 404.11, 404.90, 404.91, 581, 582, 583, 590.0, 593.6, 593.9, 753.12, 753.13, 753.14, 250.4), chronic obstructive pulmonary disease (COPD; 490-492, 493-496), chronic liver disease (including cirrhosis and hepatic decompensation; 070.2, 070.3 070.41, 070.42, 070.44, 070.51, 070.52, 070.54, 070.6, 070.7, 070.9, 573.1, 273.4, 275.0, 275.1, 453.0, 571, 573, 576.1,456.0, 456.1, 456.2, 789.5, 789.59, 572.2, 567, 572.4), atopic dermatitis (691), allergic rhinitis (477), hepatitis B virus infection (070.2, 070.3, V02.61), hepatitis C virus infection (070.44, 070.51, 070.54, 070.7, V02.62), and depression (296, 300, 309, 311).

### Statistical Analysis

Continuous variables (such as age and length of hospital stay) were transformed into ordinal variables. To evaluate the differences of study variables among IC/BPS and non-IC/BPS groups, the absolute standardized difference (ASD) was used. A small difference was defined as an ASD of <0.1. The incidence density (per 10,000 person-months) and its 95% confidence interval (95% CI) for AD was calculated by using Poisson distribution. The hazard ratio (HR) for AD with a 95% CI was estimated using the Cox proportional hazards regression model and then adjusted for age, sex, urbanization and comorbidities in a multivariable model. We used the propensity score matching to deal with the sensitivity analysis. The propensity score (predicted probability of IC/BPS exposure) was estimated for each patient using logistic regression, and the adjusted factors included age, sex, urbanization, hospital stay, and comorbidities. The paired patients with and without IC/BPS were randomly matched at a ratio of 1:4 where the difference of propensity score was closest. A significance level was set at two-tailed *P* < 0.05. Furthermore, we assessed the cumulative incidence curve using the Kaplan–Meier method and examined the difference by using the log-rank test.

## Results

A total of 1,095 patients with IC/BPS and 21,900 1:20-matched non-IC/BPS controls were selected for this study. The median follow-up time was 66 months. Table 1 presents the information on the demographic variables and comorbidities of patients with IC/BPS and paired non-IC/BPS individuals. Of patients in the study groups, 80.18% were women. In IC/BPS groups, urban residence, longer hospital stay, and comorbidities, including hypertension, DM, hyperlipidemia, CAD, CKD, COPD, chronic liver diseases, allergic rhinitis, and depression, were more prevalent. The distributions of all observed baseline characteristics between the two groups were similar after propensity score matching.

**Table 1:** Demographic variables and comorbidities of patients with IC/BPS and paired non-IC/BPS individuals.

	**Before PSM**			**After PSM**		
	**Non-IC/BPS**	**IC/BPS**	**ASD**	**Control**	**IC/BPS**	**ASD**
	***n* = 21,900**	***n* = 1,095**		***n* = 4,224**	***n* = 1,056**	
**Age at index date**			0.000			0.05
<30	3,366 (15.37%)	168 (15.34%)		636 (15.06%)	166 (15.72%)	
30–45	6,274 (28.65%)	316 (28.86%)		1,183 (28.01%)	305 (28.88%)	
45–60	6,439 (29.40%)	320 (29.22%)		1,211 (28.67%)	305 (28.88%)	
≥60	5,821 (26.58%)	291 (26.58%)		1,194 (28.27%)	280 (26.52%)	
**Sex**			0.000			0.018
Female	17,560 (80.18%)	878 (80.18%)		3,359 (79.52%)	847 (80.21%)	
Male	4,340 (19.82%)	217 (19.82%)		865 (20.48%)	209 (19.79%)	
**Urbanization**			0.116			0.037
Urban	13,386 (61.12%)	707 (64.57%)		2,678 (63.40%)	678 (64.20%)	
Sub-urban	6,339 (28.95%)	302 (27.58%)		1,200 (28.41%)	293 (27.75%)	
Rural	2,175 (9.93%)	86 (7.85%)		346 (8.19%)	85 (8.05%)	
**Length of hospital stay**			0.268			0.000
0	20,014 (91.39%)	898 (82.01%)		3,548 (84.00%)	889 (84.19%)	
1-6	1,090 (4.98%)	98 (8.95%)		339 (8.03%)	89 (8.43%)	
≥7	796 (3.63%)	99 (9.04%)		337 (7.98%)	78 (7.39%)	
**Co-morbidities**						
Hypertension	3,978 (18.16%)	244 (22.28%)	0.103	1,021 (24.17%)	233 (22.06%)	0.050
Diabetes mellitus	1,813 (8.28%)	131 (11.96%)	0.122	496 (11.74%)	120 (11.36%)	0.012
Hyperlipidemia	2,015 (9.20%)	162 (14.79%)	0.173	571 (13.52%)	149 (14.11%)	0.017
Coronary artery disease	1,201 (5.48%)	101 (9.22%)	0.144	334 (7.91%)	91 (8.62%)	0.026
Stroke	753 (3.44%)	50 (4.57%)	0.058	194 (4.59%)	47 (4.45%)	0.007
Chronic kidney disease	596 (2.72%)	79 (7.21%)	0.208	236 (5.59%)	62 (5.87%)	0.012
COPD	1,224 (5.59%)	124 (11.32%)	0.207	431 (10.20%)	112 (10.61%)	0.013
Chronic liver diseases	1,382 (6.31%)	128 (11.69%)	0.189	455 (10.77%)	112 (10.61%)	0.005
Atopic dermatitis	251 (1.15%)	25 (2.28%)	0.088	76 (1.80%)	18 (1.70%)	0.007
Allergic rhinitis	1,686 (7.70%)	125 (11.42%)	0.127	512 (12.12%)	114 (10.80%)	0.042
Hepatitis B virus infection	279 (1.27%)	27 (2.47%)	0.088	96 (2.27%)	24 (2.27%)	0.000
Hepatitis C virus infection	137 (0.63%)	9 (0.82%)	0.023	41 (0.97%)	7 (0.66%)	0.034
Depression	1,899 (8.67%)	266 (24.29%)	0.431	955 (22.61%)	228 (21.59%)	0.025

*IC/BPS, interstitial cystitis/bladder pain syndrome; ASD, absolute standardized difference; PSM, propensity-score matching; COPD, chronic obstructive pulmonary disease*.

**Figure 2:**
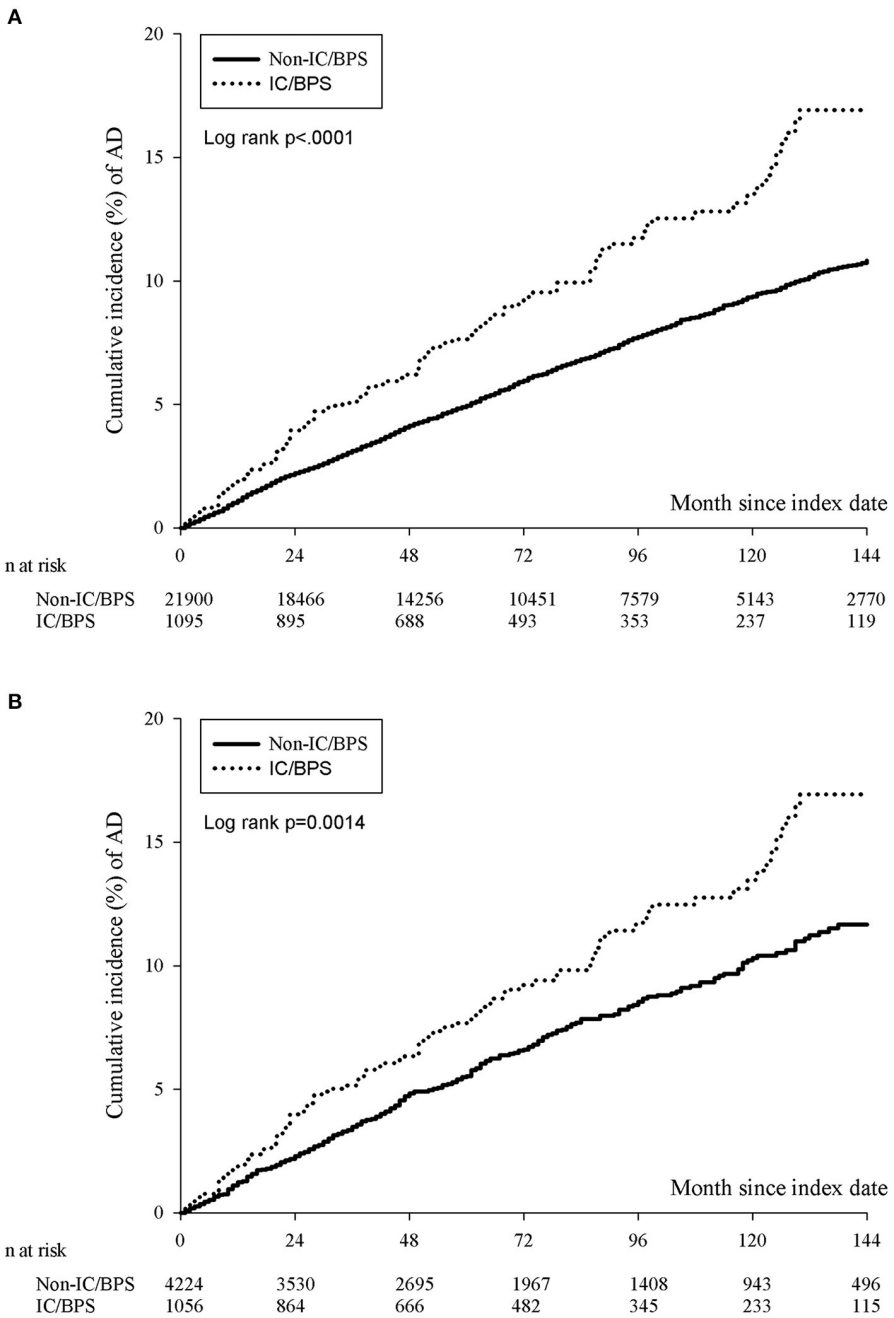
The cumulative incidence of ADs in participants with IC/BPS before **(A)** and after **(B)** PSM. The IC/BPS group was significantly higher than that in those without IC/BPS (log-rank test, *p* = 0.0014). IC/BPS, interstitial cystitis/bladder pain syndrome; ADs, autoimmune diseases; PSM, propensity score matching.

The incidence rate of ADs was evaluated before and after propensity score matching. For patients with IC/BPS, the incidence rate was 13.27 (95% CI 10.96–16.06) per 10 000 person-months, and that for controls was 8.30 (95% CI 7.87–8.75) (Table 2) for the case cohort. The crude relative risk was 1.595(95% CI 1.308–1.945). Table 3 shows the HR of ADs calculated using Cox proportional hazards regression in age- and sex-matched populations. The aHR of ADs for patients with IC/BPS was 1.409 (95% CI 1.152–1.725; *P* < 0.01). Compared with patients aged under 30 years, patients aged 31–45, 46–60, and over 60 exhibited a gradually elevating aHRs of 1.35, 1.63, and 2.60, respectively. We also observed that CKD, COPD, and depression were significantly associated with higher risk of ADs. Figure 2 shows that the cumulative incidence of ADs in participants with IC/BPS was significantly higher than in those without IC/BPS (log-rank test, *P* = 0.0014).

**Table 2:** Time to event analysis.

	**Before PSM**		**After PSM**	
	**Non-IC/BPS**	**IC/BPS**	**Control**	**IC/BPS**
	***n* = 21 900**	***n* = 1,095**	***n* = 4,224**	***n* = 1,056**
Follow up person months	1,654,806	79,147	312,164	76,839
Event of ADs	1,373	105	287	102
Incidence rate^*^ (95% CI)	8.30 (7.87–8.75)	13.27 (10.96–16.06)	9.19 (8.19–10.32)	13.27 (10.93–16.12)
Model 1: Crude hazard ratio (95% CI)	Reference	1.595 (1.308–1.945)	Reference	1.442 (1.151–1.808)

* *per 10,000 person-months*.

*IC/BPS, interstitial cystitis/bladder pain syndrome; ADs, autoimmune diseases; PSM, propensity score matching; CI, confidence interval*.

**Table 3:** Estimation of the hazard ratio of ADs by using Cox proportional hazard regression in age and sex matched population.

	**Univariate modeling**		**Multiple modeling**	
	**HR**	**95% CI**	**aHR**	**95% CI**
Exposure of IC/BPS (reference: non-IC/BPS)	1.595	1.308–1.945	1.409	1.152–1.725
**Age at index date (reference:** ** <30)**				
30–45	1.443	1.183–1.760	1.358	1.112–1.657
45–60	1.836	1.511–2.231	1.632	1.338–1.991
≥60	2.399	1.978–2.910	2.007	1.628–2.473
Sex- Male (reference: Female)	0.675	0.585–0.778	0.750	0.649–0.867
**Urbanization (reference: Urban)**				
Sub-urban	1.058	0.944–1.185	1.054	0.940–1.181
Rural	0.995	0.833–1.189	0.919	0.768–1.100
**Co-morbidities**				
Hypertension	1.469	1.298–1.662	0.986	0.849–1.146
Diabetes mellitus	1.485	1.254–1.758	1.028	0.849–1.244
Hyperlipidemia	1.538	1.313–1.801	1.125	0.941–1.344
Coronary artery disease	1.610	1.334–1.943	1.107	0.901–1.359
Stroke	1.253	0.950–1.653	0.846	0.634–1.128
Chronic kidney disease	1.915	1.504–2.439	1.451	1.128–1.866
COPD	1.621	1.353–1.942	1.252	1.036–1.512
Chronic liver diseases	1.405	1.176–1.678	1.108	0.917–1.339
Atopic dermatitis	1.412	0.927–2.151	1.262	0.827–1.925
Allergic rhinitis	1.228	1.023–1.474	1.131	0.940–1.362
Hepatitis B virus infection	1.114	0.709–1.752	0.983	0.621–1.555
Hepatitis C virus infection	1.694	0.959–2.989	1.227	0.689–2.186
Depression	1.819	1.575–2.100	1.477	1.271–1.718

*IC/BPS, interstitial cystitis/bladder pain syndrome; ADs, autoimmune diseases; HR, hazard ratio; aHR, adjusted hazard ratio; CI, confidence interval; COPD, chronic obstructive pulmonary disease*.

We present the risks of ADs in sex and age subgroups of patients with IC/BPS in Table 4. In the sex subgroup analysis, women with IC/BPS exhibited a higher risk of developing ADs (aHR = 1.427, 95% CI 1.149–1.772); for men with IC/BPS, the aHR was 1.254 (95% CI 0.708–2.223). However, the interaction between sex and IC/BPS was not significant (*P* = 0.5988). In the age subgroup analysis, those aged 45–60 exhibited a higher aHR of roughly 1.765 for developing ADs (95% CI 1.268–2.456), and no significant interaction between age and IC/BPS was noted (*P* = 0.2224).

**Table 4:** Sub-group analysis in age-sex matched population.

	Incidence rate^*^ **(95% C.I.)**		
**Sub-group**	**Non-IC/BPS**	**IC/BPS**	**aHR+ (95% CI)**
**Sex-subgroup**			
Female	8.89 (8.39–9.41)	14.52 (11.84–17.82)	1.427 (1.149–1.772)
Male	6.05 (5.29–6.93)	8.23 (4.78–14.18)	1.254 (0.708–2.223)
*p* for interaction			0.5988
**Age-subgroup**			
<30	5.01 (4.21–5.97)	4.08 (1.7–9.81)	0.679 (0.268–1.721)
30–45	6.92 (6.25–7.67)	10.79 (7.45–15.63)	1.337 (0.899–1.988)
45–60	8.68 (7.89–9.55)	18.26 (13.44–24.8)	1.765 (1.268–2.456)
≥60	11.74 (10.72–12.85)	16.76 (11.78–23.83)	1.310 (0.906–1.894)
*p* for interaction			0.2224

* *per 10,000 person months*.

*+ adjusted for demographic variables, length of hospital stay, co-morbidities at baseline*.

*IC/BPS, interstitial cystitis/bladder pain syndrome; aHR, adjusted hazard ratio; CI, confidence interval*.

To clarify the causes of ADs, we analyzed the risk of each type of AD (Table 5). Owing to limited numbers of subjects with rarer ADs, we have not presented results for multiple sclerosis, systemic sclerosis, polymyositis, Behcet's disease, or autoimmune hemolytic anemia. Compared with the control group, the IC/BPS group had a 1.516-fold (95% CI 1.001–2.296) higher risk of RA, a 1.962-fold (95% CI 1.370–2.809) higher risk of SS, a 2.429-fold (95% CI 1.264–4.67) higher risk of AS, and a 2.767-fold (95% CI 1.039–7.368) higher risk of Hashimoto's thyroiditis. Sensitivity analysis by propensity score matching with balanced sex, age, and comorbidities yielded consistent results, namely HR = 1.442(95% CI 1.151–1.808).

**Table 5:** Sub-event of ADs in study group.

	Incidence rate^*^ **(95% CI)**		
**Sub-event**	**Non-IC/BPS**	**IC/BPS**	**aHR+ (95% CI)**
**All ADs**	8.3 (7.87–8.75)	13.27 (10.96–16.06)	1.409 (1.152–1.725)
Hashimoto's thyroiditis	0.18 (0.13–0.25)	0.59 (0.24–1.41)	2.767 (1.039–7.368)
Ankylosing spondylitis	0.44 (0.35–0.55)	1.29 (0.72–2.34)	2.429 (1.264–4.67)
Myasthenia gravis	0.1 (0.07–0.16)	0.23 (0.06–0.93)	1.823 (0.407–8.166)
Rheumatoid arthritis	1.76 (1.57–1.97)	2.97 (2.01–4.4)	1.516 (1.001–2.296)
Sjogren's syndrome	1.72 (1.53–1.92)	4.18 (3–5.82)	1.962 (1.37–2.809)
Psoriasis and similar disorders	0.91 (0.78–1.06)	1.41 (0.8–2.49)	1.177 (0.645–2.149)
Polymyalgia rheumatica	0.16 (0.11–0.23)	0.35 (0.11–1.09)	1.828 (0.538–6.208)
Guillain–Barre'syndrome	0.07 (0.04–0.13)	0.12 (0.02–0.83)	1.64 (0.206–13.031)
Crohn's disease	1.28 (1.12–1.46)	1.3 (0.72–2.34)	0.94 (0.51–1.734)
Ulcerative colitis	0.17 (0.12–0.24)	0.23 (0.06–0.93)	1.336 (0.311–5.737)
Vasculitis	0.14 (0.09–0.21)	0.23 (0.06–0.94)	1.681 (0.392–7.211)
Uveitis	0.81 (0.69–0.95)	0.82 (0.39–1.72)	1.002 (0.466–2.154)
Graves' disease	0.69 (0.57–0.82)	0.35 (0.11–1.09)	0.474 (0.150–1.500)
Systemic lupus erythematosus	0.27 (0.2–0.36)	0.12 (0.02–0.83)	0.314 (0.042–2.327)
Dermatomyositis	0.05 (0.03–0.1)	0.12 (0.02–0.83)	2.939 (0.364–23.734)

* *per 10,000 person months*.

*+ adjusted for demographic variables, length of hospital stay, co-morbidities at baseline*.

*IC/BPS, interstitial cystitis/bladder pain syndrome; aHR, adjusted hazard ratio; ADs, autoimmune diseases; CI, confidence interval*.

## Discussion

In the present study, we investigated the risk of concomitant ADs among individuals with IC/BPS using a nationwide database. The main results showed that patients with IC/BPS had an increased risk of developing ADs (aHR = 1.409, 95% CI 1.152–1.725) after adjusting for age, sex, and comorbidities. The risk was most prominent in Hashimoto's thyroiditis (aHR = 2.767, 95% CI 1.039–7.368), AS (aHR = 2.429, 95% CI 1.264–4.67), RA (aHR = 1.516, 95% CI 1.001–2.296), and SS (aHR = 1.962, 95% CI 1.37–2.809). Additionally, we independently stratified the subgroups of patients with IC/BPS into specific age and sex groups and identified a higher incidence rate in female patients (aHR = 1.427, 95% CI 1.149–1.772) and patients aged 45 to 60 years (aHR = 1.765, 95% CI 1.268–2.456) after adjusting for demographic variables, length of hospital stay, and comorbid conditions. However, the risks of SLE and inflammatory bowel disease (Crohn's disease and ulcerative colitis) were not increased in our study, a finding not in concordance with previous case-control studies (7, 14). These results indicated the possible causality underlying the association of IC/BPS and diverse ADs.

Some novel findings were made in our study. First, the risk of Hashimoto's thyroiditis was the highest in patients with IC/BPS in this study. However, there are currently no existing studies indicating a possible connection between IC/BPS and Hashimoto's thyroiditis. Hashimoto's thyroiditis is an autoimmune–endocrine disorder that mistakes the thyroid for an invader and attacks the thyroid tissue with a series of antibodies (23). In thyroid diseases, Chung et al. demonstrated that patients with IC/BPS had 2.16 times the odds ratio (OR) of patients without IC/BPS but with a previous diagnosis of hyperthyroidism (16). Another nationwide population-based study noted a higher prevalence of hypothyroidism with an adjusted OR of 2.3 times in patients with IC/BPS (24). These studies support our finding that IC/BPS may increase the risk of subsequent immune-related pathogenesis in the thyroid gland. Second, a positive relationship was shown between SLE and IC/BPS in the previous studies. A cross-sectional observational study, conducted by Keller et al., reported a higher prevalence of SLE (OR = 2.57) in patients with IC/BPS (24). Another cohort study demonstrated that patients with SLE had an increased risk for IC/BPS of 2.45 times (12). However, the aHR of SLE in our study was insignificant. We deduce that the sample size of IC/BPS patients with subsequent occurrence of SLE was still too small to provide enough statistical power to be statistically significant in our study.

In clinical practice, several major ADs such as SS, RA, and AS are pivotal and have drawn much attention for investigation by specialists. With regard to SS, patients showed a significantly increased risk of IC/BPS (HR = 2.34) in a large study involving 11,526 cases (10). As for RA, a case-control study showed that patients with IC/BPS and a previous diagnosis of RA had an OR of 1.66; however, men had a higher OR than women in their sex stratification (11), a finding contrasting with the female predominance seen in our results. AS has also been reported to be more prevalent in patients with IC/BPS (24). However, the specific mechanisms which underlie this relationship are still unknown. According to previous research, the pathogenesis between IC/BPS and ADs was induced by the release of specific cytokines evoked by autoantibodies transported to the lamina propria of the urinary bladder (10). Furthermore, relevant case reports have revealed abnormal laboratory tests with increased IgG, decreased C4, and antinuclear antibodies (ANA) in patients with SS presenting IC/BPS-related symptoms (25, 26). Merwe et al. determined that the antimuscarinic receptor IgG, an antagonist of the bladder and salivary and lachrymal glands, might play an important role in the early and late stages of the development of IC/BPS (27). Consequently, IC/BPS is likely to be a preceding local expression of origin participating in the immune responses of systemic ADs. As evidenced cited above, some studies have considered AD to be one of the causes of IC/BPS. However, further research is still required to verify whether IC/BPS triggers diverse ADs through any specific mechanism.

In our study, women and middle-aged patients had a higher risk of ADs. In previous research, the strong female preponderance has often been noted as a possible indicator of the autoimmune nature of IC/BPS (2). According to a study, the average age of onset of major ADs in women is as follows: SLE, age 15–55; RA, age 30–60; SS, age 40–60 (28). Therefore, middle-aged women are predisposed to present symptoms of IC/BPS and ADs due to their similar age of onset.

The strengths of our study are as follows. First, the use of a longitudinal and large-scale population database enables the results to be generalized up to a nationwide level. This not only minimizes the selection biases inherent in the investigation of the risks of ADs in patients with IC/BPS with various characteristics but also tracks a follow-up period of sufficient duration to allow for dysfunction to manifest in the urinary bladder. By contrast, previous studies have tended to analyze single AD and thus lack such a comprehensive review in their summaries. Second, this study examined the incidence risk of ADs in relation to chronic exposure to IC/BPS by utilizing various methods of matching analysis, including propensity score matching, and univariate- and multiple modeling to corroborate the results. Nevertheless, the present findings must be interpreted with caution due to several limitations. First, diagnoses from the NHIRD through ICD- 9-CM codes were concerned with miscoding or misclassification, and may thus be less accurate than standardized criteria. However, we further applied the same methods of the enrollment used in previous studies to improve the diagnostic validity. Second, the study design could not exclude the surveillance bias of patients with IC/BPS, especially middle-aged women, seeking health care services more often, thus resulting in a heightened tendency to receive medical examinations and be diagnosed with ADs. Third, although we have included many ADs to yield a generalized exploration in this study, some diseases with a potential relationship to autoimmunity might not yet be covered. Fourth, the confounding effects of comorbidities, such as health information and dietary habits and other environmental variables, still could not be completely avoided. However, we performed propensity score analysis to provide additional validity. Fifth, the NHIRD database was available until 2013 during the period of our IRB application, thus the trend in recent years was unable to be evaluated. However, our study has already included a 13-year cohort with adequate representation. Sixth, despite the large sample size in our study, the included population was still limited to a nation-base, which might influence the generalizability of the results to the worldwide population.

In conclusion, IC/BPS was associated with the development of ADs in patients in Taiwan's NHIRD research, especially in those with Hashimoto's thyroiditis, AS, RA, and SS. To date, no existing studies indicate a possible connection between IC/BPS and Hashimoto's thyroiditis. Clinicians are recommended to raise consensus on the patients with a history of IC/BPS and be alert to the increased likelihood of developing ADs, particularly for middle-aged women. Further studies are needed to confirm our findings and explore the underlying pathological mechanisms.

## Data Availability Statement

The original contributions presented in the study are included in the article/supplementary material, further inquiries can be directed to the corresponding author.

## Ethics Statement

This study was approved by the Institutional Review Board of Chung Shan Medical University (IRB, CS15134).

## Author Contributions

H-ZY, M-HY, and J-YH were accountable for study conception and design and acquisition of data. J-YH performed the analyses and helped interpret the results. H-ZY and M-HY wrote the original draft of the manuscript. JW was involved in the supervision and revision of the manuscript. All authors have read, provided critical revision on intellectual content, and approved the final version of the manuscript.

## Funding

The Longitudinal Health Insurance Database 2000 (LHID2000) was purchased from Taiwan National Health Insurance Administration by Chung Shan Medical University Hospital (Grant No. CSMU-INT-104-04).

## Conflict of Interest

The authors declare that the research was conducted in the absence of any commercial or financial relationships that could be construed as a potential conflict of interest.

## Publisher's Note

All claims expressed in this article are solely those of the authors and do not necessarily represent those of their affiliated organizations, or those of the publisher, the editors and the reviewers. Any product that may be evaluated in this article, or claim that may be made by its manufacturer, is not guaranteed or endorsed by the publisher.
